# A specific plasminogen activator inhibitor‐1 antagonist derived from inactivated urokinase

**DOI:** 10.1111/jcmm.12875

**Published:** 2016-05-20

**Authors:** Lihu Gong, Valerie Proulle, Chao Fang, Zebin Hong, Zhonghui Lin, Min Liu, Guangpu Xue, Cai Yuan, Lin Lin, Barbara Furie, Robert Flaumenhaft, Peter Andreasen, Bruce Furie, Mingdong Huang

**Affiliations:** ^1^ State Key Laboratory of Structural Chemistry and Danish‐Chinese Centre for Proteases and CancerFujian Institute of Research on the Structure of MatterChinese Academy of SciencesFuzhouFujianChina; ^2^ University of Chinese Academy of SciencesBeijingChina; ^3^ Division of Hemostasis and ThrombosisBeth Israel Deaconess Medical CenterHarvard Medical SchoolBostonMAUSA; ^4^ Department of Molecular Biology and GeneticsAarhus UniversityAarhus CDenmark

**Keywords:** PAI‐1, urokinase variants, protein structure, fibrinolysis, antithrombotic agent

## Abstract

Fibrinolysis is a process responsible for the dissolution of formed thrombi to re‐establish blood flow after thrombus formation. Plasminogen activator inhibitor‐1 (PAI‐1) inhibits urokinase‐type and tissue‐type plasminogen activator (uPA and tPA) and is the major negative regulator of fibrinolysis. Inhibition of PAI‐1 activity prevents thrombosis and accelerates fibrinolysis. However, a specific antagonist of PAI‐1 is currently unavailable for therapeutic use. We screened a panel of uPA variants with mutations at and near the active site to maximize their binding to PAI‐1 and identified a potent PAI‐1 antagonist, PAItrap. PAItrap is the serine protease domain of urokinase containing active‐site mutation (S195A) and four additional mutations (G37bR–R217L–C122A–N145Q). PAItrap inhibits human recombinant PAI‐1 with high potency (*K*_d_ = 0.15 nM) and high specificity. *In vitro* using human plasma, PAItrap showed significant thrombolytic activity by inhibiting endogenous PAI‐1. In addition, PAItrap inhibits both human and murine PAI‐1, allowing the evaluation in murine models. *In vivo,* using a laser‐induced thrombosis mouse model in which thrombus formation and fibrinolysis are monitored by intravital microscopy, PAItrap reduced fibrin generation and inhibited platelet accumulation following vascular injury. Therefore, this work demonstrates the feasibility to generate PAI‐1 inhibitors using inactivated urokinase.

## Introduction

As a member of the serpin family, PAI‐1 is a plasma inhibitor of enzymes involved in fibrinolysis, including urokinase (uPA) and tissue plasminogen activator (tPA). Plasminogen is activated by uPA or tPA to generate plasmin, an enzyme that lyses fibrin in thrombi. After thrombus formation to protect vessel integrity following tissue injury in the high‐pressure circulatory system, patency of the vessel is re‐established by the lysis of fibrin in the clot. PAI‐1 inhibits plasminogen activators rapidly and irreversibly, and is the primary negative regulator of the fibrinolytic system.

Given thrombosis as the leading cause of death in Western society, significant attention has been given to the development of therapeutic agents for both inhibition of thrombus formation and promotion of fibrinolysis. These agents can be used to treat acute myocardial infarction, stroke, arterial thrombi and venous thromboembolic events, including pulmonary embolism. The current clinical approach has been to infuse exogenous plasminogen activators, such as tPA or urokinase, into either the local or systemic circulation to lyse fibrin within formed thrombi 1.

Endogenous plasminogen activators play an important physiological role in maintaining a patent vascular system. Given that inhibition of PAI‐1 augments the activities of the endogenous plasminogen activators, PAI‐1 has been recognized as a potential therapeutic target for fibrinolytic treatment of thrombotic disorders 2, 3. Many laboratories, including ours 4, have developed PAI‐1 antagonists, which include small molecules 5, 6, 7, 8, 9, 10, monoclonal antibodies 11, 12, peptides 13 and RNA aptamers 14. Despite the efforts to develop PAI‐1 antagonists, none have entered clinical use. The major challenges include attainment of both high specificity and potency, and capability to inhibit both human and murine PAI‐1, because mice models are typically used for antagonist evaluation.

In this work, we describe a strategy for the development of a new type of PAI‐1 antagonist. This antagonist, based on the natural PAI‐1 ligand urokinase (uPA), is enzymatically inactive and engineered to bind tighter to PAI‐1 compared to the inactivated uPA. As such, it has both high specificity and high affinity for PAI‐1. We call this optimized urokinase analogue PAItrap. PAItrap shows fibrinolytic properties in plasma *in vitro. In vivo*, using a laser‐induced mouse arteriolar thrombosis model, PAItrap reduced fibrin generation and inhibited platelet accumulation following vascular injury.

## Materials and methods

### Mice

Wild‐type C57BL/6J mice and PAI‐1‐deficient mice (B6.129S2‐Serpine1tm1Mlg) 15, 16 were obtained from Jackson Laboratory.

### Reagents

Platelets were imaged *in vivo* using anti‐CD42 antibodies conjugated with Dylight 488 (Emfret Analytics, Eibelstad, Germany). Fibrin was detected *in vivo* using a mouse anti‐human fibrin monoclonal antibody (clone 59D8), labelled with Alexa 647, that cross‐reacts with mouse fibrin but not fibrinogen 17. Full‐length tPA was from Boehringer Ingelheim, Ingelheimam Rhein, Germany. Antithrombin‐III, α‐thrombin, human α_2_‐antiplasmin and human plasmin were from Haematologic Technologies, Essex Junction, USA. PN‐1 was a gift from Denis Monard, Friedrich Mischer Institute, Basel, Switzerland. Aprotinin was purchased from Fischer Scientific, Pittsburgh, USA. The chromogenic substrate *H*‐d‐pyroglutamyl‐Gly‐l‐Arg‐*p*‐nitroanilide (S‐2444) and Gly‐Arg‐p‐nitroanilide were from Chromogenix Instrumentation Laboratories, Milan, Italy and Sigma‐Aldrich, St. Louis, USA respectively. Human blood was collected in citrated tubes from healthy donors who were informed about the objectives of the study. Platelet‐rich plasma was prepared by centrifugation at 100 × *g* for 10 min at room temperature. Aliquots of platelet‐rich plasma were centrifuged at 1500 × *g* for 10 min to obtain platelet‐poor plasma and the supernatant plasma was centrifuged at 11,000 × *g* for 5 min to obtain platelet‐free plasma.

### Cloning and expression

The uPA serine protease domain (uPA‐SPD) domain, lacking the amino terminal fragment of uPA, served as the structural base for PAItrap. The structure of the isolated SPD of uPA is indistinguishable from the SPD of full‐length active uPA 18. The cDNA for active‐site‐mutated uPA in which serine 195 is mutated to alanine (uPA‐S195A) was expressed in the vector pPICZaA (Invitrogen, Carlsbad, USA). Any additional mutations in this cDNA were carried out using the Quick Change II site‐directed mutagenesis kit (Agilent, Santa Clara, USA). All mutations were confirmed by DNA sequencing. Recombinant uPA‐S195A variants were then expressed using the yeast X‐33 (EasySelect Pichia Expression Kit; Invitrogen) according to the manufacturer's recommendations. Recombinant variants were purified from expression medium by a cation exchange column SPFF and eluted with a NaCl gradient (0–1 M) in 20 mM phosphate buffer, pH 6.5. The eluent was concentrated in a Millipore ultrafiltration tube and then applied to a Superdex 75 HR 10/30 gel filtration column equilibrated with 20 mM phosphate buffer, pH 6.5, 150 mM NaCl. Recombinant human uPA‐SPD and a stable PAI‐1 variant (named 14‐1B) 19 were expressed and purified as described 4, 18.

Recombinant human PAI‐2 (expression vector kindly provided by Marie Ranson, University of Wollongong, Australia) was expressed in bacterial M15 cells and purified according to the published method 20.

### Assay of PAI‐1 inhibitory activity

A chromogenic assay was used to measure PAI‐1 activity as measured by its inhibition of uPA‐dependent hydrolysis of peptide substrates. Human recombinant PAI‐1 was pre‐incubated with increasing concentrations of uPA‐S195A variants for 10 min followed by the addition of human uPA. After 10 min, a chromogenic substrate, S‐2444, was added to the mixture. The final reaction contained 20 mM Tris pH 7.4, 150 mM NaCl, 0.05% Tween‐20, varying concentrations of uPA‐S195A variants, 15 nM PAI‐1, 15 nM uPA and 80 μM S‐2444. The remaining uPA activity was measured by the initial rate of cleavage of S‐2444 at 405 nm. The potency of uPA‐S195A variants was determined by the increase of uPA activity that was initially inhibited by PAI‐1. All experiments were performed three times.

### Inhibitory effects of PAItrap on other serpin protease interaction

Recombinant human PAI‐2 or PN‐1 (final concentrations 15 nM) was pre‐incubated with increasing concentrations of PAItrap for 10 min at 37°C in 70 μl in 20 mM Tris pH 7.4, 150 mM NaCl, 0.05% Tween‐20, followed by the addition of 10 μl of uPA to 15 nM and further incubated for 10 min at 37°C. The remaining uPA activity was determined by incubation with S2444 (final concentrations 80 μM) and the measurement of the increase in absorbance at 405 nm at 37°C.

Human antithrombin‐III (final concentrations 30 nM) was pre‐incubated with increasing concentrations of PAItrap for 10 min at 37°C in 70 μl of 50 mM Tris‐HCl, pH 7.4, 150 mM NaCl, 0.05% Tween 20, followed by the addition of 10 μl of human α‐thrombin (final concentrations 30 nM) and further incubation for 10 min at 37°C. The remaining thrombin activity was determined using Gly‐Arg‐p‐nitroanilide at 250 μM (final concentrations) at 37°C.

The inhibition of interaction between human α_2_‐antiplasmin (final concentrations 15 nM) and human plasmin (final concentrations 15 nM) by PAItrap was also determined in parallel using 300 μM Gly‐Arg‐p‐nitroanilide. All experiments were performed three times.

### Surface plasmon resonance

Anti‐PAI‐1 monoclonal antibody (Mab‐1) (20 μg/ml) was immobilized on a CM5 Biacore chip. PAI‐1 (20 mM) was added to the chip, and the uPA‐S195A variants, ranging from 25 nM to 48.8 pM, were then applied. All experiments were repeated three times. The *k*_on_, *k*_off_ and *K*_d,_ were determined by analysing the kinetic data by a global fit to a 1:1 binding model with the BIA evaluation software (Biacore, GE, Fairfield, USA).

### Thermal stability measurements

Thermal denaturation of the PAI‐1:uPA‐S195A and PAI‐1:PAItrap complexes by circular dichroism was performed on a JASCO810 circular dichroism system equipped with Peltier temperature‐controlled cuvette holder. The signal at 222 nm was monitored while the 1 cm cuvette containing 0.1 mg/ml complex was heated in 1°C steps from 35 to 75°C. Melting point temperatures (*T*_m_) were obtained from the inflection point of the sigmoid curves by nonlinear fit of the data.

### Crystal structure of PAItrap

The crystallization of PAItrap was carried out using the method similar to that previously reported 18. With the sitting‐drop vapour‐diffusion method, crystals of the PAItrap were obtained by equilibrating against a solution containing 2.0 M ammonium sulphate, 50 mM sodium citrate (pH 4.6), and 5% PEG 400 at room temperature. The crystals appeared in about 3 days. Prior to X‐ray data collection, the crystals were soaked in a cryoprotectant solution for 5 min containing 2.2 M ammonium sulphate, 5% PEG 400, 50 mM sodium citrate (pH 4.6) and 20% glycerol, and flash‐frozen in a liquid nitrogen stream. X‐ray diffraction data of the crystals were collected at the BL17U1 beam line, Shanghai Synchrotron Radiation Facility. All diffraction data were indexed and integrated by the HKL2000 program package 21. The structure was determined by molecular replacement method using MOLREP 22 and refined using REFMAC of the CCP4 suite 23.

### Assay of PAI‐1 inhibitory activity *via* clot lysis

Clot lysis in human platelet‐poor plasma was performed as described previously 10. Recombinant PAI‐1 was pre‐incubated for 10 min at 37°C in the presence or absence of PAItrap or uPA‐S195A in 150 mM NaCl, 20 mM Tris pH 7.4, 0.05% Tween 20, with a final concentration of 0.9 nM PAI‐1, 6.5 nM uPA‐S195A or PAItrap, 2.4 nM tPA and 50% v/v plasma in a total volume of 100 μl. Clot formation was initiated by the addition of CaCl_2_ to 11 mM and clot lysis was monitored at 37°C by measuring the absorbance at 405 nm. All experiments were performed three times.

### Intravital microscopy

Intravital widefield microscopy of the cremaster muscle microcirculation was performed as described previously 24, 25 with modifications. Digital images were captured with a C9300‐201 CCD digital camera (Hamamatsu) connected to a VS4‐1845 GEN III image intensifier (VideoScope, Miami, USA) or an Orca Flash 4.0v2 CMOS camera (Hamamatsu, Shimokanzo, Japan). The Olympus microscope includes a Yokogawa CSU‐X1 A1 confocal scanner. The laser source, housed in an acousto‐optical tunable filter launch (Intelligent Imaging Innovations), includes three solid‐state lasers (Cobolt and Coherent). Vessel injury was induced with a Micropoint Laser System (Photonics) 24. Image analysis was performed with Slidebook Version 5.5 (Intelligent Imaging Innovations, Denver, USA) 25.

### Platelet accumulation and fibrin generation during thrombus formation

Platelet accumulation and fibrin generation were evaluated *in vivo* using a rat IgG against the GPIbβ subunit of the murine GPIb‐V‐IX complex labelled with Dylight 649 to identify platelets and anti‐fibrin‐Alexa 488 to visualize fibrin. Platelet thrombus size and fibrin generation were determined by calculating area under the curves for the median values of the fluorescence at 649 or 488 nm *versus* time. Median platelet and fibrin fluorescence curves were derived from 22 to 40 thrombi per group generated in at least two mice per group over the course of approximately 150 sec.

### Statistical analysis

The Mann–Whitney test was used for statistical comparison of the areas under the curves for platelet and fibrin fluorescence between two groups of animals. Linear regression analysis was performed for the dose curves of ERp5 binding to αIIbβ3 and for the time course of ERp5 release from HUVEC. Statistical analyses were performed with Prism software package (version 5.02; GraphPad, La Jolla, USA). *P* values of 0.05 or less were considered statistically significant and are indicated.

### Vertebrate animal and human experimentation

The BIDMC Institutional Animal Care and Use Committee approved animal care and procedures. The collection of human blood samples was approved by the Institutional Review Board of Fujian Institute of Research on the Structure of Matter, Chinese Academy of Sciences.

## Results

### Design of a novel PAI‐1 antagonist

**Figure 1:**
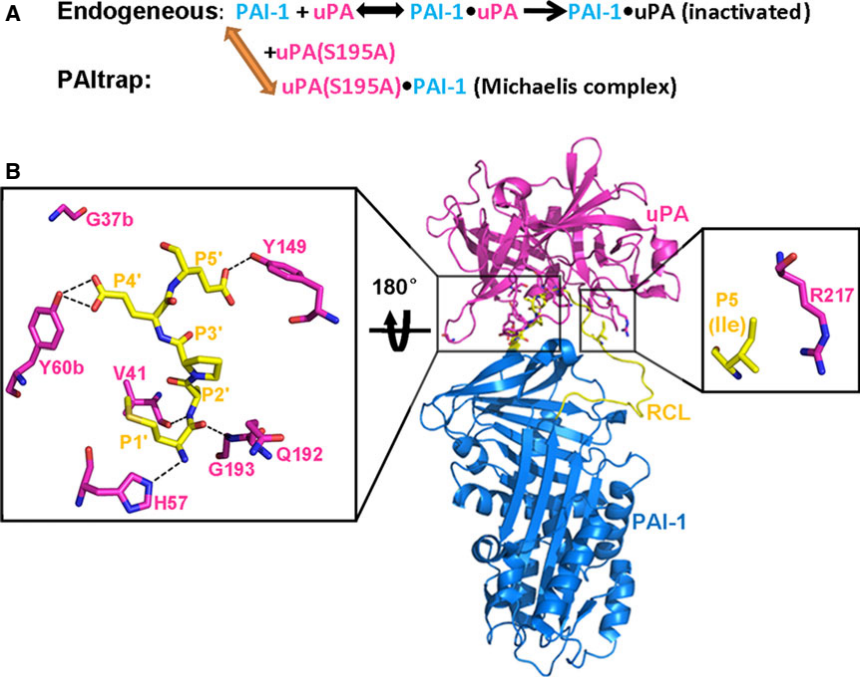
Design of active‐site‐mutated uPA (uPA‐S195A) variants as a PAI‐1 antagonist (PAItrap). Active‐site‐mutated uPA (uPA‐S195A) can trap PAI‐1 in Michaelis complex, but can uPA‐S195A compete with endogenous uPA which forms a covalent complex with PAI‐1 (**A**)? In (**B**): Crystal structure of PAI‐1•uPA‐SPD‐S195A Michaelis complex (middle: PDB code 3PB1) 28 revealed that the binding interface between PAI‐1 and uPA‐S195A is not energetically optimal. A variant with a basic amino acid residue at uPA Gly37b or Tyr60b has potential to form charged hydrogen bonds to P4’ residue (Glu350) (Left). A variant with leucine at uPA Arg217 promotes hydrophobic interaction with P5 residue (Ile342) (Right). These modifications were hypothesized to improve the binding between the uPA variants and PAI‐1. uPA (magenta); PAI‐1 (cyan); reactive centre loop (yellow).

Given the high specificity of urokinase for PAI‐1, we used the uPA‐SPD as a template to engineer a potent inhibitor of PAI‐1. uPA‐S195A does not possess proteolytic activity, but can bind to PAI‐1 and form a Michaelis complex (Fig. 1).

Although uPA‐S195A can function as a PAI‐1 antagonist 26, uPA‐S195A cannot compete effectively with the endogenous plasminogen activators for PAI‐1. uPA‐S195A binds to PAI‐1 less tightly than endogenous uPA because the active‐site serine residue is important for binding to the substrate or inhibitor 27. In addition, the endogenous uPA cleaves PAI‐1 and forms a covalent and irreversible complex with PAI‐1, while the uPA‐S195A forms non‐covalent complex with PAI‐1. Thus, it will be preferred if the uPA‐S195A can be further engineered to have stronger binding to PAI‐1 than wild‐type uPA.

We previously determined the crystal structure of the Michaelis complex between PAI‐1 and uPA‐S195A 28. The structure revealed that the binding interface between PAI‐1 and uPA‐S195A is not energetically optimal (Fig. 1). Therefore, we engineered the uPA‐S195A to optimize its binding to PAI‐1 18, 28. We used the following principles to introduce mutations on uPA‐S195A as PAI‐1 antagonists:

1. Reverse charge mutations to promote formation of charged hydrogen bonds. Hydrogen bonds between opposite charged residues are much stronger (4.5 kcal/mol) than regular hydrogen bonds (2–3 kcal/mol) 29.
2. Point mutations to promote hydrophobic interaction between uPA mutants and PAI‐1. Hydrophobic interactions are mediated by van der Waal forces and are the major driving force for protein folding.

Using these two principles, we chose seven uPA‐S195A mutations: Y60bK, G37bR, R217L, Y151F, Q192K, Y149R and A96E. We generated these uPA variants together with three additional mutations: S195A to render uPA enzymatically inactive; C122A to prevent potential uPA‐SPD homodimerization; N145Q to eliminate an *N*‐glycosylation site. uPA variants (11) (Table 1) were expressed in yeast cells and purified by ion exchange chromatography and gel filtration on Superdex G75.

**Table 1:** IC_50_s of uPA‐SPD variants on PAI‐1 measured by the chromogenic assay

uPA‐SPD variants	IC_50_ (nM)
S195A‐C122A‐N145Q	109 ± 24
S195A‐C122A‐N145Q‐G37bR	25 ± 2
S195A‐C122A‐N145Q‐Y60bK	39 ± 2
S195A‐C122A‐N145Q‐A96E	22 ± 3
S195A‐C122A‐N145Q‐Y149R	>3000
S195A‐C122A‐N145Q‐Y151F	~1000
S195A‐C122A‐N145Q‐Q192K	~3000
S195A‐C122A‐N145Q‐R217L	48 ± 5
S195A‐C122A‐N145Q‐G37bR‐R217L (PAItrap)	10 ± 1
S195A‐C122A‐N145Q‐G37bR‐Y60bK	90 ± 10
S195A‐C122A‐N145Q‐G37bR‐Y60bK‐R217L	26 ± 2
S195A‐C122A‐N145Q‐G37bR‐R217L‐A96E	30 ± 2

### Optimization of PAI‐1 inhibition by mutation of uPA‐S195A

**Figure 2:**
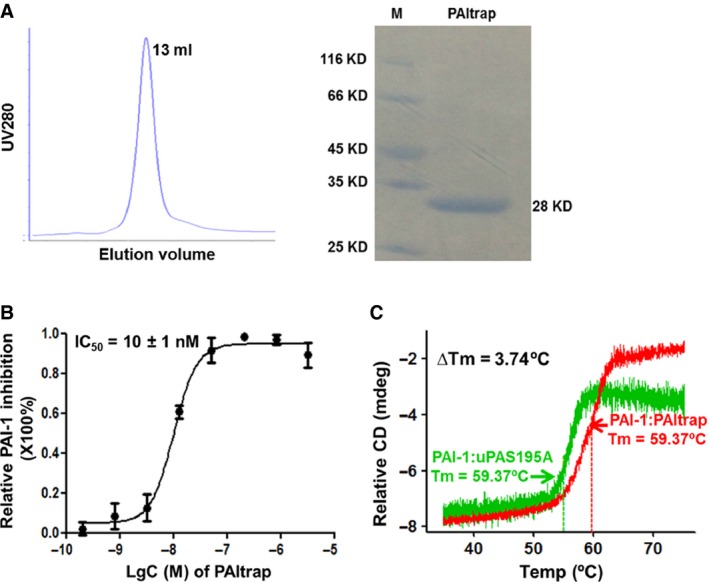
Properties of PAItrap (uPA‐S195A‐G37bR‐R217L‐C122A‐N145Q). (**A**) PAItrap purified by Superdex 75 gel filtration (left) was examined by SDS‐PAGE (right). (**B**) PAItrap inhibits PAI‐1 activity, yielding an IC_50_ of 10 nM. The IC_50_ was measured by monitoring the effect of PAItrap on inhibition of PAI‐1 during urokinase hydrolysis of S‐2444 in the presence of PAI‐1. (**C**) The dissociation of PAI‐1:PAItrap complex as a function of temperature was monitored by circular dichroism at 222 nm. Melting temperature of the PAI‐1:PAItrap complex is ~4°C higher than the PAI‐1:uPA‐S195A complex.

The IC_50_ values of all variants are presented in Table 1. Single mutations of G37bR, Y60bK, R217L or A96E, in addition to S195A‐C122A‐N145Q, yielded more potent PAI‐1 inhibition with lower IC_50_ values compared to that of uPA‐S195A (IC_50_ 109 nM). Other mutations (Y149R, Y151F or Q192K) increased the IC_50_. Next, we combined the effective single mutations to generate double mutations. The G37bR‐R217L double variant had the lowest IC_50_ (10 nM, Fig. 2B), which is about 10‐fold lower than uPA‐S195A, and is named as PAItrap. The incorporation of other mutations (e.g. Y60bK or A96E) into PAItrap did not further optimize potency (IC_50_ of 26 and 30 nM respectively). We also measured the ability of PAItrap in blocking PAI‐1 inhibition on tPA, yielding an IC_50_ of 14 nM (see Fig. S1).

### Direct binding of uPA‐S195A variants to PAI‐1

We further evaluated the uPA‐S195A variants by measuring their binding to PAI‐1 by surface plasmon resonance. PAI‐1 was bound to a monoclonal anti‐PAI‐1 antibody immobilized on a Biacore chip, and the uPA‐S195A variants were applied to the chip. The binding kinetics for the variants to the bound PAI‐1 were studied, and the *k*_on_, *k*_off_ and *K*_d_ were calculated (Table 2). The uPA‐S195A variants with G37bR, Y60bK, R217L, G37bR‐Y60bK and G37bR‐R217L (PAItrap) mutations showed lower *K*_d_ values against PAI‐1 than against uPA‐S195A. The PAItrap variant, G37bR‐R217L, had the highest affinity for PAI‐1, with a *K*_d_ of 0.15 nM. The trend of these data is parallel to the IC_50_ values determined by activity measurements.

**Table 2:** On‐rate, off‐rate and dissociation constants (*K*_d_) of uPA‐SPD variants from PAI‐1 measured by SPR

uPA‐SPD variants	*k*_on_ (10^5^/Ms)	*k*_off_ (10^−4^/s)	*K*_d_ (nM)
S195A‐C122A‐N145Q	7.76 ± 0.60	12.1 ± 0.5	1.57 ± 0.20
S195A‐C122A‐N145Q‐G37bR	23.0 ± 5.4	6.98 ± 0.32	0.32 ± 0.06
S195A‐C122A‐N145Q‐Y60bK	17.0 ± 2.4	6.20 ± 0.25	0.37 ± 0.06
S195A‐C122A‐N145Q‐R217L	13.3 ± 0.4	8.40 ± 0.56	0.63 ± 0.03
S195A‐C122A‐N145Q‐G37bR‐Y60bK	23.5 ± 16.1	11.4 ± 4.2	0.83 ± 0.53
S195A‐C122A‐N145Q‐G37bR‐R217L	41.1 ± 2.0	6.27 ± 1.02	0.15 ± 0.02

The binding of PAItrap from surface plasmon measurement (*K*_d_ 0.15 nM) is much tighter than that obtained from the chromogenic substrate‐based assay using active uPA (IC_50_ 10 nM), although both these assays identified PAItrap as the best variant in the PAI‐1 inhibition. This difference likely reflects the fact that PAItrap forms a reversible complex with PAI‐1, whereas uPA forms an irreversible complex with PAI‐1. The *K*_*d*_ value involves only the reversible binding between PAItrap and PAI‐1 and reflects the PAItrap concentration with 50% of PAI‐1 bound. By contrast, the IC_50_ value is the PAItrap concentration where 50% of uPA is enzymatically active, and is affected not only by the reversible binding of PAItrap on PAI‐1, but also by the conditions used in the assays, for example, uPA concentration.

### Properties of PAItrap (uPA‐S195A‐G37bR‐R217L‐C122A‐N145Q)

PAItrap, a protein with a molecular weight of about 28,000, was purified to near homogeneity as shown by gel filtration and SDS‐PAGE (Fig. 2A). The dose‐dependent inhibition of PAI‐1 activity by PAItrap yielded an IC_50_ value for PAI‐1 inactivation of 10 nM (Fig. 2B). We further measured the thermal denaturation of PAItrap in complex with PAI‐1 by monitoring changes in the circular dichroism spectra at 222 nm (Fig. 2C). Its melting temperature (*T*_m_ 59.37°C) was about 4°C higher than that of the PAI‐1:uPA‐S195A complex (*T*_m_ 55.63°C), confirming that PAItrap indeed binds tighter to PAI‐1 compared to uPA‐S195A.

**Figure 3:**
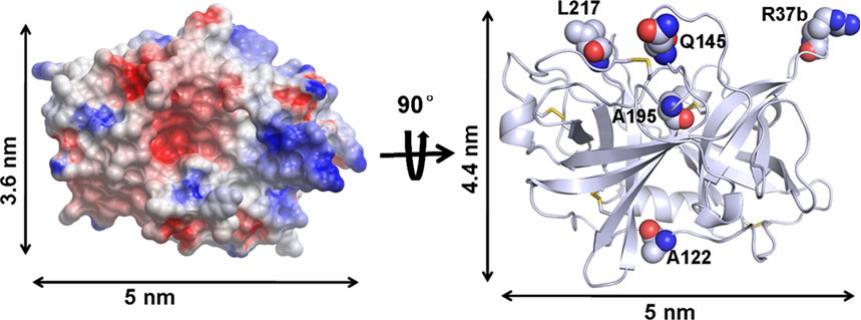
X ray crystal structure of PAItrap at 1.5 Å. PAItrap is a globular protein as shown by its crystal structure at 1.5 Å. The charged surface representation is shown on the left and a ribbon diagram is presented on the right. PAItrap is an engineered urokinase with five residue substitutions including glycine 37b to arginine, cysteine 122 to alanine, asparagine 145 to glutamine, serine 195 to alanine and arginine 217 to leucine. These substitutions are shown as CPK models. The chymotrypsin numbering system is employed.

PAItrap was crystallized and its crystal structure was determined to 1.5 Å (Fig. 3 and Table 3). The structure shows that the mutations introduced to uPA did not affect the overall protease fold, based on its high structural similarity to wild‐type uPA with a root‐mean‐square‐deviation of 0.15 Å for all Cα atoms. The structure further shows that PAItrap is a globular protein with negatively charged concave surface (Fig. 3), a site that interacts with PAI‐1. The coordinates for the PAItrap structure have been deposited in the Protein Data Bank (http://www.rcsb.org/pdb/) under the accession number 4XSK.

**Table 3:** X‐ray data collection and model refinement statistics for PAItrap crystals

Data collection statistics	
Space group	R3
Cell dimensions	*a* = *b* = 121.03 Å, *c* = 42.77 Å, α = β = 90.00°, γ = 120.00°
Wavelength, Å	0.979
Resolution, Å	34.94–1.5 (1.55–1.5)
<I/σ(I)>	27 (2.4)
Completeness (%)	99.82 (99.71)
Multiplicity	3.8 (3.7)
*R*_merge_	0.099
*Model refinement statistics*	
R‐work/R‐free	0.174 (0.185)/0.209 (0.220)
RMS deviations of bonds (Å)/angles (°) from ideality	0.023/2.219
% residues in most favoured/outlier regions in Ramachandran plot	95.5%/1.6%
Average B‐factor (Å^2^) of model	18

Numbers in parentheses are for the highest resolution shell.

### Specificity of PAItrap for PAI‐1

**Figure 4:**
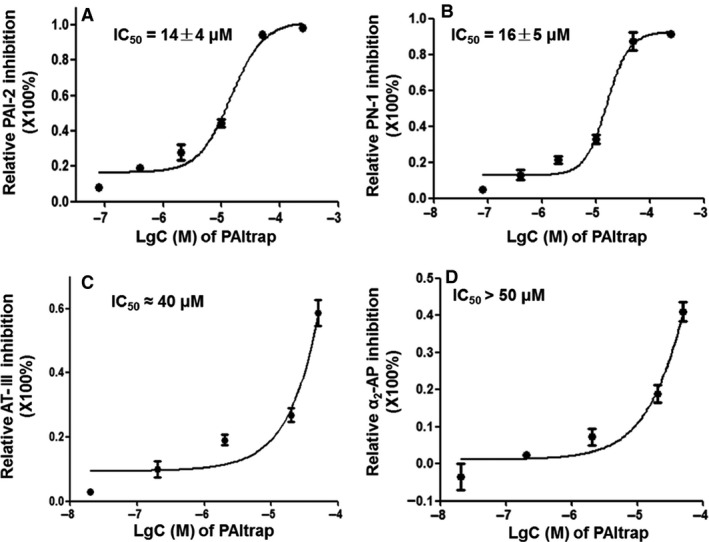
Specificity of PAItrap for PAI‐1. The inhibition of serpins by PAItrap in comparison to PAI‐1. The ratio of PAItrap inhibition of the serpin studied compared to PAI‐1 is presented as a function of PAItrap concentration. (**A**) PAI‐2; (**B**) PN‐1; (**C**) anti‐thrombin‐III; (**D**) 2‐antiplasmin.

uPA activates plasminogen and can be inhibited by several serpins other than PAI‐1, which include plasminogen activator inhibitor‐2 (PAI‐2), PN‐1, antithrombin‐III and α2‐antiplasmin. We measured PAItrap inhibition to this list of serpins, which gave IC_50_s of 14 ± 4 μM, 16 ± 5 μM, 40 μM and >50 μM respectively (Fig. 4). All these values were at least 1400‐fold greater than that of the IC_50_ for PAI‐1 (10 ± 1 nM), demonstrating a very high selectivity of PAItrap to PAI‐1.

### PAItrap promotes fibrinolysis *in vitro*

**Figure 5:**
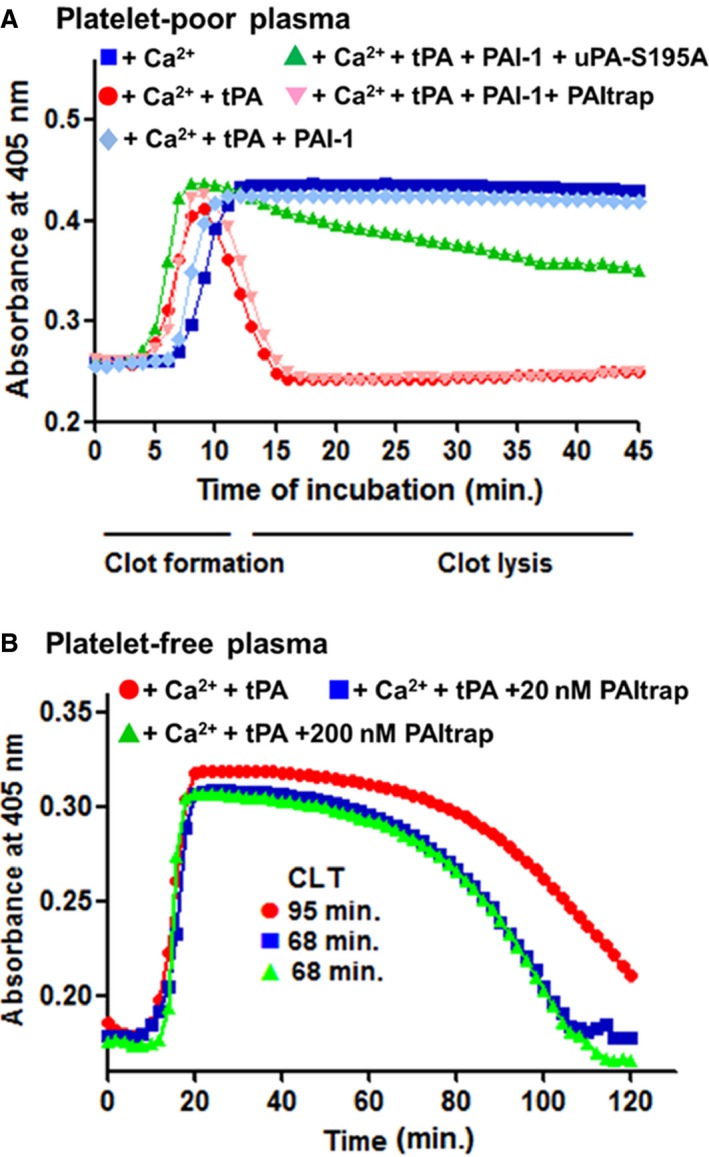
PAItrap promotes fibrinolysis in plasma *in vitro*. (**A**) The kinetics of change in clot turbidity in human platelet‐poor plasma was monitored at 405 nm following the addition of tPA. Calcium alone, ■, dark blue; tPA, ●, red; tPA+PAI‐1+PAItrap, ▼, pink; tPA+PAI‐1+uPA‐S195A, ▲, green; tPA+PAI‐1, ♦, light blue. (**B**) Clot lysis assay using platelet‐free plasma in the absence of exogenous PAI‐1. tPA, ●, red; tPA+20 nM PAItrap, ■, blue; tPA+200 nM PAItrap, ▲, green. Shown is a representative experiment of three independent experiments.

The effect of PAItrap on fibrinolysis initiated by tPA *in vitro* was analysed. Citrated platelet‐poor plasma was pre‐incubated with tPA (to 2.4 nM), clotted by the addition of calcium ions, and the clot formation and lysis were monitored for up to 45 min at OD 405 nm (Fig. 5A) on a plate reader. Under these conditions, the clot was lysed in 6 min. The addition of PAI‐1 (0.9 nM) abolished the effects of tPA and no clot lysis was observed. In the presence of uPA‐S195A (6.5 nM), clot lysis was modestly increased because of its neutralization of PAI‐1 inhibition of tPA. In contrast, PAItrap (6.5 nM) added to plasma in the presence of tPA facilitated clot lysis, with complete lysis in approximately 6 min. This accelerated lysis clear demonstrates that a mere 10‐fold increase of the potency of PAItrap over uPA‐S195A translates to significant differences in clot lysis effect.

To further evaluate whether PAItrap inhibits endogenous plasma PAI‐1, we generated fibrin clots without the addition of exogenous PAI‐1 and with variable concentrations of PAItrap (Fig. 5B). The addition of tPA to 100 pM promoted 50% clot lysis in 96 min in the assay using platelet‐free plasma, whereas the further inclusion of 20 nM of PAItrap in the assay accelerated lysis to 68 min. PAItrap (20 nM) appears to neutralize this pool of circulating PAI‐1 because a higher dose of PAItrap (200 nM) did not further shorten the lysis time.

### Inhibition of thrombus formation by PAItrap in a mouse thrombosis model

**Figure 6:**
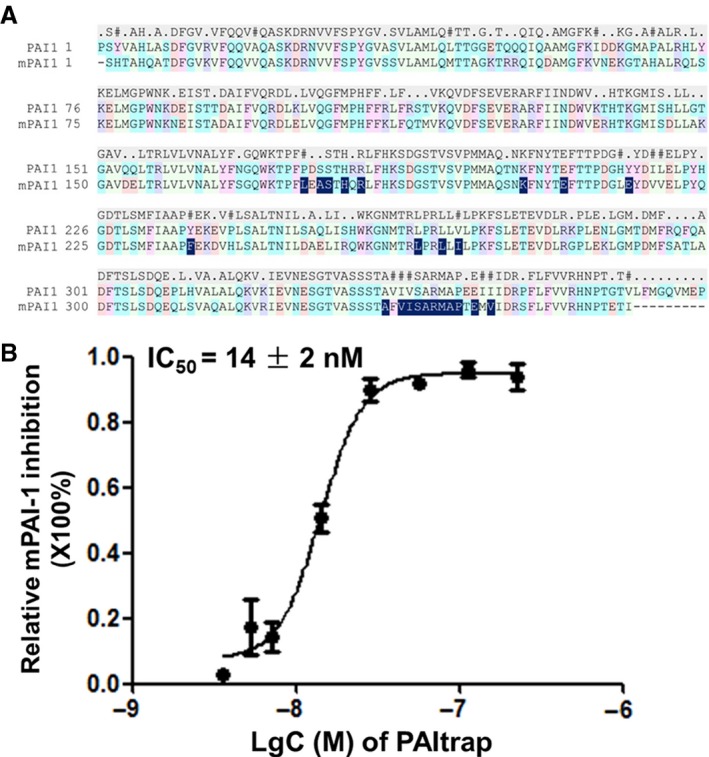
PAItrap inhibits mouse PAI‐1. (**A**) Aligned sequences of human and murine PAI‐1. The highlighted residues in dark shadows are the PAI‐1 residues that are in contact with uPA modelled based on PDB entry 3PB1. These uPA‐contacting residues are mostly conserved between human and murine PAI‐1 sequences, suggesting that PAItrap designed based on human uPA should be able to inhibit murine PAI‐1. (**B**) PAItrap inhibited murine PAI‐1 with an IC_50_ of 14 nM based on the chromogenic assay similar to that used for human PAI‐1.

**Figure 7:**
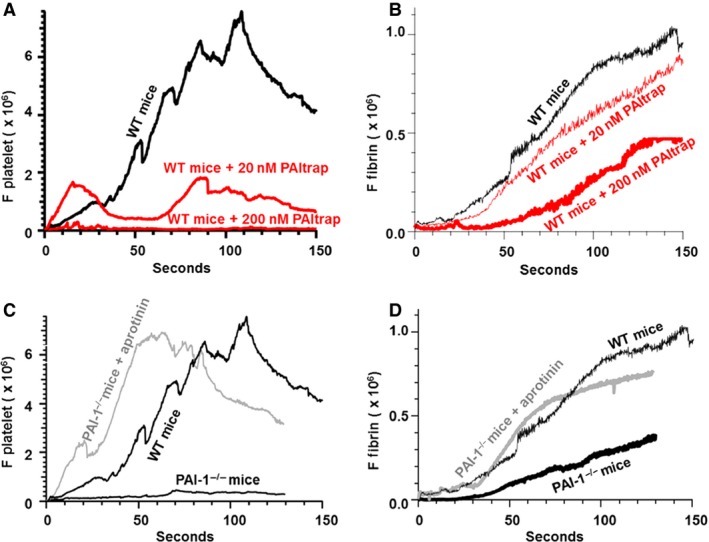
*In vivo* thrombus formation in a mouse model of arteriolar laser‐induced injury before and after treatment with PAItrap. (**A**) Kinetics of the fluorescence associated with platelet accumulation (F Platelet) before (black; 35 thrombi, 3 mice) and after infusion of 20 nM (thin red; 21 thrombi, 2 mice) or 200 nM (thick red; 25 thrombi, 4 mice) PAItrap. (**B**) Kinetics of the fluorescence associated with fibrin accumulation (F Fibrin) before (black; 35 thrombi, 3 mice) and after infusion of 20 nM (thin red; 21 thrombi, 2 mice) or 200 nM (thick red; 25 thrombi, 4 mice) PAItrap. Effect of aprotinin, a plasma inhibitor. (**C**) Kinetics of the fluorescence associated with platelet accumulation (F Platelet) before (black; 34 thrombi, 4 mice) and after (grey; 18 thrombi, 2 mice) infusion of aprotinin (40,000 Kallikrein Inhibitor Unit/kg) in PAI‐1‐null mice. The kinetics of platelet accumulation in wild‐type mice is also shown (thick black; 35 thrombi, 3 mice). (**D**) Kinetics of the fluorescence associated with fibrin accumulation (F Fibrin) before (thick black; 34 thrombi, 4 mice) and after (grey; 18 thrombi, 2 mice) infusion of aprotinin (40,000 Kallikrein Inhibitor Unit/kg) in PAI‐1‐null mice. The kinetics of fibrin accumulation in wild‐type mice is also shown (thin black; 35 thrombi, 3 mice).

Fibrinolytic proteins show significant differences between human and mouse 30, posing challenges for the evaluation of human modulators in mouse models sometimes. We showed that the PAItrap developed here inhibited murine PAI‐1 with an IC_50_ (14 nM, Fig. 6B) comparable to that for human PAI‐1. This is because of the unique strategy used for PAItrap: PAItrap recognizes a set of PAI‐1 residues that happen to be identical between human and murine PAI‐1 (Fig. 6A). Based on this result and the reasonable stability of PAItrap in mice (see Supplemental Information), we evaluated the effect of PAItrap in an *in vivo* mouse thrombosis model in which vascular injury was induced by laser injury to an arteriole and thrombus formation monitored by intravital microscopy. Platelet accumulation and fibrin generation were imaged during thrombus formation for 150 sec. PAItrap was infused into the mouse circulation to a final plasma concentration of 20 or 200 nM and thrombus formation kinetics was monitored. Platelet accumulation and fibrin generation were quantified by calculating the area under the curves for fluorescence associated with platelets and fibrin. PAItrap almost eliminated platelet accumulation and decreased fibrin generation by about 50% at 20 and 200 nM PAItrap compared to thrombus formation prior to PAItrap infusion (Fig. 7A and B).

To confirm that inhibition of PAI‐1 leads to inhibition of platelet accumulation as well as fibrin generation, we performed similar experiments in PAI‐1‐null mice (Fig. 7C and D). Compared to wild‐type mice, PAI‐1‐deficient mice showed minimal platelet accumulation and marked decrease in the amount of fibrin present. Thus, the thrombus formation kinetics in PAI‐1‐null mice recapitulated that observed in the PAItrap‐treated mice.

Although decreased fibrin accumulation was anticipated as a result of the inhibition of PAI‐1 or absence of PAI‐1 since fibrinolysis would be enhanced by increased endogenous plasminogen activators, the absence of platelet accumulation could not be explained by increased endogenous plasminogen activators. To explain this, we hypothesized that decreased platelet accumulation in PAI‐1‐null mice or PAI‐1‐inhibited mice might be because of excessive enhancement of plasmin activity. The elevated local plasmin activity in PAI‐1‐null or PAI‐1‐inhibited mice may inhibit platelet aggregation through proteolytic inactivation of platelet surface receptors. To test this hypothesis, we infused an inhibitor of plasmin, aprotinin (40 inhibitor unit/g), into the circulation of PAI‐1‐null mice. Platelet thrombus formation in PAI‐1‐null mice and fibrin generation were completely rescued after infusion of aprotinin (Fig. 7C and D), thus supporting this hypothesis.

## Discussion

Efforts to develop suitable inhibitors of PAI‐1 have been complicated by the critical need for high specificity for PAI‐1. Low molecular weight compounds lack this specificity and binding affinity. Development of protein inhibitors based on a natural ligand such as urokinase (with catalytic serine residue mutated to typically alanine, uPA‐S195A) by exploiting its specificity for PAI‐1 was proposed before 26, but has been complicated by the fact that endogenous urokinase forms a covalent complex with PAI‐1, whereas an active‐site‐mutated urokinase interacts non‐covalently. Here, we have performed a series of amino acid substitutions on the modified urokinase rationally based on the crystal structure of the urokinase:PAI‐1 Michaelis complex. This structure showed that the interaction between PAI‐1 and uPA‐S195A is not energetically optimal. This allowed us to design and screen a series of uPA‐S195A variants to enhance their PAI‐1 inhibitory capability, and led to a novel protein inhibitor of PAI‐1, which we call PAItrap. PAItrap exhibits 10‐fold tighter binding to PAI‐1 (*K*_d_ 0.15 nM) compared to uPA‐S195A (*K*_d_ 1.6 nM). This engineered protein retains the specificity necessary and bind sufficiently tightly that is in the range for potential clinical use. Although PAItrap is only 10‐fold more potent than uPA‐S195A, it accelerated the clot lysis from 39 min of uPA‐S195A to about 6 min at the identical concentration (6.5 nM). In addition, the PAItrap appeared to block the thrombus formation efficiently in live mice, without impacting the tail bleeding time of mice, one indicator for global haemostasis (see in Fig. S2).

The functional data in this study suggest that PAItrap might be efficacious to prevent thrombus formation. Furthermore, we cannot rule out that PAItrap might be a useful adjunct to the administration of thrombolytic agents by lowering the dose of thrombolytics necessary for fibrinolysis.

PAItrap is derived from inactivated human protease. Although there are five mutations which may render this protein immunogenic to the host, most of the structure is native and 98% of the amino acid sequence is identical to the urokinase SPD. If the PAItrap would be moved to clinical application, the immunogenicity after the repeated usage of PAItrap would need to be evaluated.

## Disclosures

The authors confirm that there are no conflicts of interest.

## Author contributions

L.G.: idea development, preliminary experiments, experiments, data analysis and manuscript preparation; V.P.: mouse experiments and data analysis; C.F.: mouse experiments and data analysis; Z.H.: idea development, preliminary experiments and experiments; Z.L.: idea development and preliminary experiments; M.L.: experiments; G.X. and C.Y.: experiments; L.L.: mouse experiments; B.C.F. and R.F.: data analysis; P.A.: design of study and manuscript preparation; B.F.: data analysis and manuscript preparation; M.H.: design of study, data analysis and manuscript preparation.

## Supporting information


**Figure S1** PAItrap blocks PA‐1 inhibition on tPA, yielding an IC_50_ of 14 nM.
**Figure S2** Effect of PAItrap on global hemostasis of mice.
