# Supplementary material for: A specific plasminogen activator inhibitor‐1 antagonist derived from inactivated urokinase
Source: J Cell Mol Med. 2016 May 20;20(10):1851–60. doi: 10.1111/jcmm.12875 (PMC4876229; doi:10.1111/jcmm.12875)
Supplement: Supplementary file 1 — Figure S1 PAItrap blocks PA‐1 inhibition on tPA, yielding an IC50 of 14 nM. Figure S2 Effect of PAItrap on global hemostasis of mice. [file JCMM-20-1851-s001.doc]

**SUPPLEMENTAL INFORMATION**

**FOR**

**A specific Plasminogen activator inhibitor-1 Antagonist DERIVED from inactivated urokinase**

Lihu Gong^1,4#^, Valerie Proulle^2#^, Chao Fang^2#^, Zebin Hong^1^, Zhonghui Lin^1^, Min Liu^1,4^, Guangpu Xue^1^, Cai Yuan^1^, Lin Lin^2^, Barbara Furie^2^, Robert Flaumenhaft^2^, Peter Andreasen^3^, Bruce Furie^2^, Mingdong Huang^1,2,4*^

^1^State Key Laboratory of Structural Chemistry and Danish-Chinese Centre for Proteases and Cancer, Fujian Institute of Research on the Structure of Matter, Chinese Academy of Sciences, Fuzhou, 350002, Fujian, China

^2^Division of Hemostasis and Thrombosis, Beth Israel Deaconess Medical Center, Harvard Medical School, 330 Brookline Ave., Boston, MA 02215, USA

^3^**Department of Molecular Biology and Genetics, A**arhus University, 8000 Aarhus C, Denmark

^4^University of Chinese Academy of Sciences, Beijing, 100049, China

^#^Equal contributing authors.

***Correspondence to Dr. Mingdong Huang**, E-mail: mhuang@fjirsm.ac.cn

**PAItrap blocks PAI-1 inhibition on tPA-**A chromogenic assay was used to measure PAI-1 activity on its inhibition of tPA-dependent hydrolysis of peptide substrates. Human recombinant PAI-1 was preincubated with increasing concentrations of PAItrap for 10 min, on a 96-well microplate, followed by the addition of human tPA. A chromogenic substrate, Gly-Arg-­p-­nitro­anilide, was then added to the mixture, giving a solution of 20 mM Tris pH 7.4, 150 mM NaCl, 0.05% Tween-20, varying concentrations of PAItrap, 15 nM PAI-1, 15 nM tPA, and 200 µM Gly-Arg-­p-­nitro­anilide. The remaining tPA activity was measured by the initial rate of cleavage of Gly-Arg-­p-­nitro­anilide at 405 nm. The potency of PAItrap was determined by the increase of tPA activity that was initially inhibited by PAI-1. The IC50 for tPA inhibition turned out to be 14 nM, comparable to PAItrap inhibition to uPA.


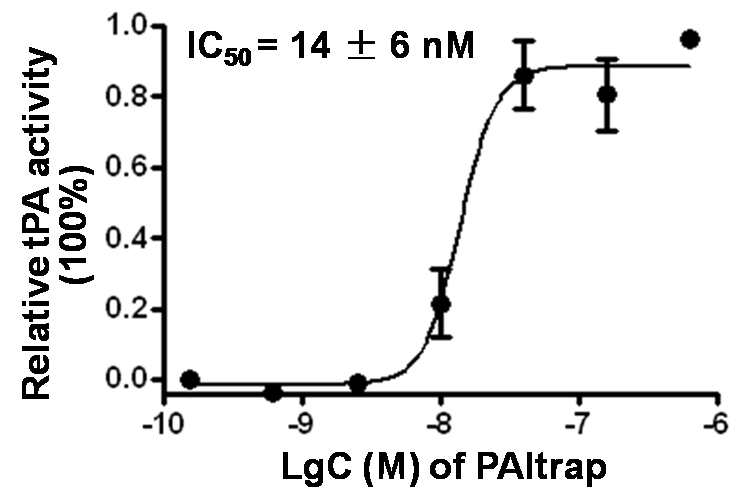


Fig. S1. **PAItrap blocks PAI-1 inhibition on tPA, yielding an IC_50_ of 14 nM**.

**Stability of PAItrap *in vivo*-**PAItrap was injected into mice through the tail vein to a plasma concentration of 10 μM. We used this high concentration of PAItrap in the experiment in order to accurately measure the remaining PAItrap. We collected mouse blood 30 min after PAItrap infusion. The results showed that there was still 265 nM of PAItrap left after 30 min in the circulation.

**Effect of PAItrap on the tail bleeding time in mice and stability of PAItrap *in vivo***-The tail bleeding times were measured on wild type mice 30 minutes after PAItrap infusion at a plasma concentration of 200 nM. Mice receiving PAItrap showed bleeding times and blood loss volumes comparable to untreated mice (Fig.S1), suggesting PAItrap may not affect global hemostasis of mice.


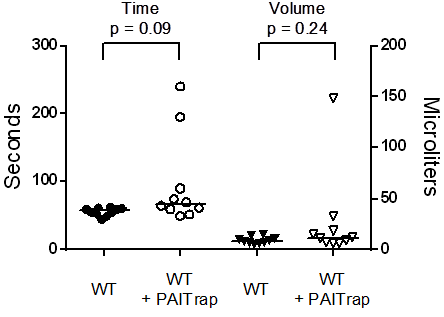


Fig. S2. **Effect of PAItrap on global hemostasis of mice**. Tail bleeding time in wild type mice before (n=10, closed circles) and after (n=10, open circles) PAItrap infusion (200 nM). B. Blood volume loss following tail excision in wild type mice before (n=10, closed triangles) and after (n=10, open triangles) PAItrap infusion. p > 0.05: non significant.
